# Optical Identification of Middle Ear Infection

**DOI:** 10.3390/molecules25092239

**Published:** 2020-05-09

**Authors:** Alisha Prasad, Syed Mohammad Abid Hasan, Manas Ranjan Gartia

**Affiliations:** Department of Mechanical and Industrial Engineering, Louisiana State University, Baton Rouge, LA 70803, USA; aprasa9@lsu.edu (A.P.); shasan4@lsu.edu (S.M.A.H.)

**Keywords:** middle ear infection, bacterial infection, otitis media, otoscope, drug-resistant microbial, label-free method, Raman spectroscopy

## Abstract

Ear infection is one of the most commonly occurring inflammation diseases in the world, especially for children. Almost every child encounters at least one episode of ear infection before he/she reaches the age of seven. The typical treatment currently followed by physicians is visual inspection and antibiotic prescription. In most cases, a lack of improper treatment results in severe bacterial infection. Therefore, it is necessary to design and explore advanced practices for effective diagnosis. In this review paper, we present the various types of ear infection and the related pathogens responsible for middle ear infection. We outline the conventional techniques along with clinical trials using those techniques to detect ear infections. Further, we highlight the need for emerging techniques to reduce ear infection complications. Finally, we emphasize the utility of Raman spectroscopy as a prospective non-invasive technique for the identification of middle ear infection.

## 1. Introduction

Ear infection or otitis media (OM) is a commonly found illness in children under the age of three [1,2,3,4,5]. The healthcare cost due to ear infections in the U.S. is estimated to be $3–5 billion annually [6,7,8]. There are three types of OM, acute otitis media (AOM), otitis media with effusion (OME), and chronic otitis media with effusion [9,10,11,12,13]. AOM occurs in the middle ear due to bacterial infection resulting in the buildup of fluid. OME also results in the buildup of fluid in the middle ear area due to inflammation and is much more severe than AOM. The symptom of both AOM and OME is the swelling of the tympanic membrane (TM). This makes it difficult to diagnose AOM from OME clinically. The typical diagnostic is based on pneumatic otoscopy, which is the gold standard to differentiate AOM from OME [14]. The pneumatic otoscope measures the deflection of TM under pressure by creating an air-tight seal. The response will change when there is fluid buildup behind the TM. In addition to the complicated process of creating an air-tight seal, the pneumatic otoscope can neither identify the bacteria involved in the infection nor distinguish viral infections from bacterial infections. Failure to differentiate viral and bacterial infections and/or AOM from OME may lead to the prescription of antibiotics, leading to drug-resistant microorganisms. Therefore, accurate detection and identification of bacteria responsible for OM is a clinically unmet need.

The three most common bacteria that are responsible for AOM and OME are *Streptococcus pneumoniae* (Gram-positive) [15,16], *Haemophilus influenza* (Gram-negative), and *Moraxella catarrhalis* (Gram-negative) [17,18,19,20,21]. Humans asymptomatically harbor these bacteria, and they primarily colonize in the upper respiratory tract and scantily populate the lower respiratory tract. These bacteria normally act as symbiotic partners, which ultimately protect the host from getting colonized by possibly more pathogenic or antibiotic-resistant organisms. Fluid buildup in the middle ear with a moist and warm environment provides ready access and a well-suited place for bacterial growth and is the main cause of AOM [20]. Acute mastoiditis is another type of bacterial infection resulting from untreated acute otitis media, prompting serious exertion and intrusion of the mastoid cells in the mastoid bone located behind the ear [22]. In current clinical settings, Gram staining is a routine procedure followed, wherein ear swabs are collected for analysis of the bacteria. The major drawback associated with Gram staining is that although it helps in bacterial classification, it gives no information about the bacterial strain. This misinformation may cause the bacterial infection to recur as the prescribed antibiotics might be resistant to the infection. In a recent study, it was found that only in 25% of the cases, a specific bacteria stain was found in the culture; however, in nearly 80% of the same cases of OME, the specific DNA-RNA of the pathogens were accurately detected by polymerase chain reaction (PCR) method [19]. This suggest that the combination of molecular biology techniques (like analysis of DNA, RNA, protein) is needed in addition to the microbiology techniques (bacterial staining) for accurate examination of the pathogen. Although these techniques are useful, the main drawbacks include the requirement of specialized expensive equipment, highly skilled lab personnel, and time-consuming sample preparation. 

**Figure 1:**
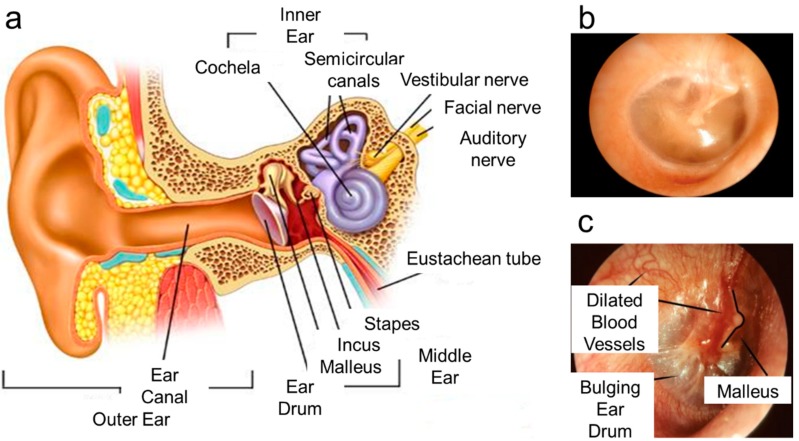
(**a**) Anatomy of a human ear. Adopted and modified from [9]. (**b**) Healthy human ear. **(c**) Infected human ear. Adopted and modified from [23], Copyright Microbiology Society, 2015.

Although most infections are a result of bacterial invasion, there are a few ear infections such as vestibular neuronitis, infectious myringitis, and herpes zoster of the ear, which are caused by viruses. Vestibular neuritis is caused by the inflammation of the vestibular nerve, which is situated at the inner ear (Figure 1a). Acute mastoiditis is a type of bacterial infection resulting from untreated acute otitis media, prompting serious exertion and intrusion of the mastoid cells in the mastoid bone located behind the ear [22]. As seen from Figure 1b, the eardrum of a healthy ear looks pinkish gray, whereas the otitis media infected middle ear in Figure 1c has a red, bulging eardrum.

In this manuscript, we reviewed the current and emerging optical techniques useful for the detection of ear infection, and in Table 1, we list them in detail. However, in some cases, the sensitivity and specificity information for ear infection were not found. However, we included the performance parameters for those cases based on the same technology that has been applied in similar fields. Accurate identification of bacteria will provide physicians additional information to prescribe the proper treatment plan while avoiding antimicrobial drug resistance. We also highlighted the Raman spectroscopy-based method as a label-free technique with minimal sample preparation to detect and identify bacteria relevant to OM.

## 2. Clinical Imaging Techniques for the Identification of Otitis Media

**Figure 2:**
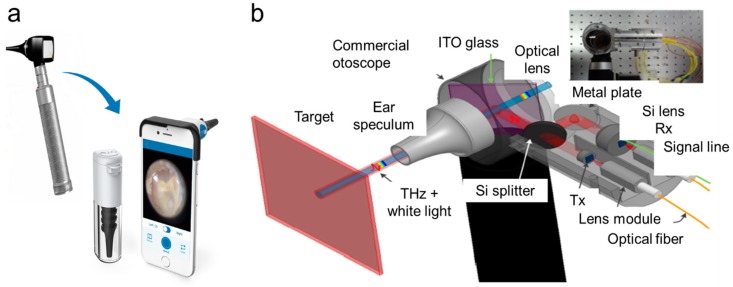
Clinically available tools to detect middle ear infection. (**a**) CellScope; (**b**) terahertz otoscope [47].

Clinically, an otoscope is the most widely used device by an otolaryngologist for identification of problems associated with ear pain. The key parameters examined in the middle ear include coloration, transparency, and the presence of liquid in the tympanic membrane [14]. In most cases, patients are advised oral or topical antibiotics; although being sufficient for pain, the infection recurs due to the evolution of antimicrobial-resistant bacteria. It has been found that the frequent use of antibiotics and bacterial biofilms can help to increase antimicrobial resistance [44,45]. Recently, Yang et al. developed an antibiotic-encapsulated hydrogel with chemical permeation enhancers to improve the drug efficiency and antimicrobial therapy of the hydrogel, when applied to the tympanic membrane through the external auditory canal of the ear. The formulated hydrogel successfully eradicated AOM from the *Haemophilus influenzae* bacteria in a chinchilla model [46]. A single dosage of such drugs would cover a complete antibiotic course, improving patient compliance, avoiding antibiotic over-dosage, and ultimately reducing recurrent side effects. Figure 2a (left) shows a typical otoscope, and Figure 2a (right) shows an iPhone-enabled otoscope (Cell Scope). Monroy et al. mentioned that an otoscope showed approximately 70% sensitivity and specificity for diagnosis of otitis media [24]. Although both the otoscope and CellScope are useful tools, visual inspection alone may not be sufficient to detect the specific type of infection, in more severe cases. AOM is associated with inflammatory changes, such as variations in the mucous membrane, accumulation of liquid, and pus. Some visual observation can be made with the otoscope; however, any chemical changes or identification of bacterial phenotype are not possible with a conventional otoscope. Ji et al. designed and constructed a terahertz (THz) otoscope, which works on THz technology or in other words electromagnetic waves to detect water molecules sensitively for feasible AOM diagnosis. The detection of electromagnetic waves enabled by the terahertz technology is helpful for obtaining precise ear membrane information. Figure 2b shows the schematics of a THz otoscope, which is integrated with a conventional optical otoscope with a fiber-coupled THz module. It was interesting to see that this device can be used as both a THz otoscope, as well as a conventional otoscope. While THz technology has been used in several biomedical applications, this technology helps the physician diagnose AOM as well. From their results, it was evident that it was possible to identify the presence of water located at the membrane and sample interface with respect to different types of THz pulses regulated by membrane thickness and separate refractive indices [47]. 

A few other clinical methods for the diagnosis of otitis media and otitis media-related infections include acoustic reflectometry, tympanometry, and pneumatic otoscopy. Acoustic reflectometry is used to measure the fluid formation in the middle ear and has 63.6–96% sensitivity and 79.7–87% specificity [31,32]. Tympanometry is used to examine the movement of the eardrum by air pressure and has 70–91% sensitivity and 71.7–98% specificity [32,33,34]. Pneumatic otoscopy is used to examine the mobility in the tympanic membrane with diagnostic accuracy (percent of total test items correct) of about 76%. Pneumatic otoscopy showed 24% improvement in sensitivity and 42% improvement in specificity, compared to a conventional otoscope [25]. However, Sundvall et al. reported the sensitivity and specificity of pneumatic otoscopy as 94% and 80%, respectively [27,35]. According to a clinical chronic ear survey (CES), it was found that in nearly 25–50% of cases of ear inspection, otolaryngologists do not use pneumatic otoscopes, while the ergonomic survey reveals that in most of the remaining cases, nearly 43% of pneumatic diagnosis are misinterpreted and performed by untrained personnel [48]. However, these three techniques—pneumatic otoscopy, acoustic reflectometry, and tympanometry—have a common limitation, as they cannot provide complete depth-resolved statistics regarding the structural variations at the tympanic membrane. Furthermore, if any kind of biofilm has formed behind the tympanic membrane, it will not be detected properly by these techniques [49].

## 3. Preclinical Imaging Techniques for the Identification of Otitis Media

**Figure 3:**
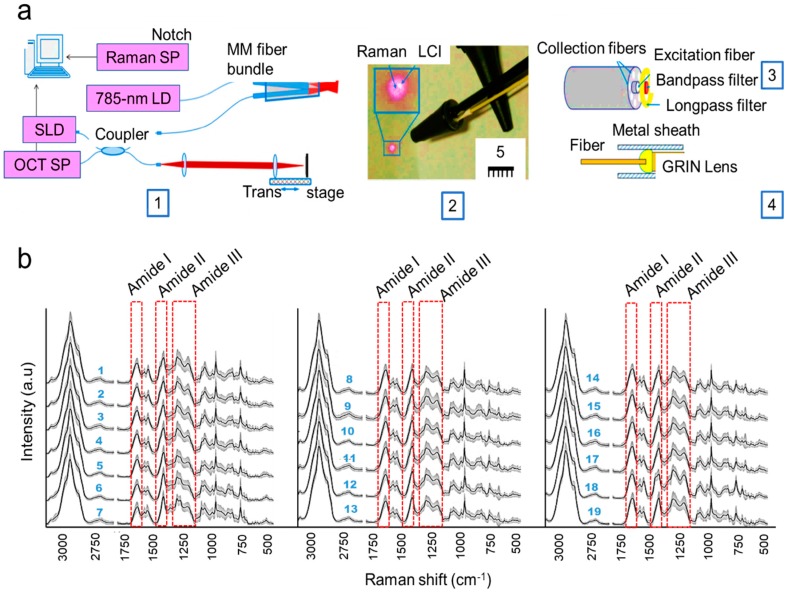
(**a**) Raman and low-coherence interferometry (Raman LCI) ((gradient index (GRIN); spectrometer (SP); laser diode (LD); superluminescence diode (SLD); multimode (MM)). Reproduced with permission from [56]. (**b**) Mean Raman spectra of common bacteria of ear infection. 1: *Listeria grayi*, 2: *Listeria innocua*, 3: *Listeria monocytogenes*, 4: *Listeria welshimeri*, 5: *Staphylococcus aureus*, 6: *Staphylococcus cohnii*, 7: *Staphylococcus epidermidis*, 8: *Escherichia coli*, 9: *Pseudomonas aeruginosa*, 10: *Pseudomonas putida*, 11: *Pseudomonas stutzeri*, 12: *Salmonella enterica*, 13: *Salmonella typhimurium*, 14: *Yersinia aldovae*, 15: *Yersinia bercovieri*, 16: *Yersinia enterocolitica*, 17: *Yersinia mollaretii*, 18: *Yersinia rohdei*, 19: *Yersinia ruckeri*. Adopted and modified with permission from [64]. Copyright Elsevier Ltd., 2013.

Optical-based diagnostics, which utilize the properties and behavior of light to obtain information about the structure and properties of matter, is emerging as a powerful technique for various biomedical examinations. Although there are a few scientific publications exploring optical imaging for middle ear evaluation, in clinical settings, they have generally been unexplored. To date, no technique manages to replicate or surpass otoscopic clinical capabilities. In this section, we cover published reports on optical imaging of middle ear pathologies. For example, Raman spectroscopy is a label-free method that utilizes the light scattering phenomena to determine the unique chemical fingerprints of any molecule by probing individual chemical bond vibrations [50]. According to the literature, the Raman spectrometer has been utilized extensively for identifying single bacteria cells or bacterial colonies based on the acquired Raman spectra [51,52,53,54]. Recently, several researchers have also exploited Raman spectroscopy for identification and detection of pathogens in ear infection cases. For example, in a recent study, a Raman spectrometer was used to understand the middle ear pathology and analyze the molecular peaks from the collected spectra [55]. They found the sensitivity and specificity to be 95.48% and 99.06%, respectively, from their experiment. However, one of the limitations of the Raman spectrometer is the weak signal, and by enhancing the signal, detection sensitivity can be improved [56]. Zhao et al. also developed a combinatorial platform assembling low-coherence interferometry (LCI) along with Raman scattering spectroscopy (RS) to identify pathogens relevant to ear infection. Figure 3a shows a schematic representation of their assembly. The LCI helps to distinguish and detect depth-resolved structural evidence of the pathogens, while the Raman spectrometer provides non-invasive molecular evidence. Together, they help to improve the diagnostic capability for identification of middle ear infection (or AOM) in real-time. On the other hand, this technology cannot detect bacteria pathogens in body fluid as it requires them to be restricted to a limited area of the microscope [56]. In another similar study, Rebrosova et al. utilized a Raman spectrometer to detect *Staphylococcal* species (n = 16) from bacterial colonies. Such studies could be applied for the identification of the *Staphylococcal* type of bacteria found prominently in ear infection patients [57,58,59,60,61,62]. Raman spectroscopy is a challenging technique, as the Raman spectra are generated from numerous molecular constituents of the target. Hence, the global spectral features of a single bacteria or bacterium would ideally show peaks within a certain range for biomolecules such as proteins (amide bonds), lipids, carbohydrates, and nucleic acids (polypeptide backbone) [63]. This information is shown in Figure 3b, and the Raman band assignments of these common bacteria of ear infection are tabulated in Table 2. 

These Raman peaks are helpful in identifying the pathogen; however, classifying these pathogenic strains as per the nomenclature is challenging without statistical analysis. Therefore, researchers have used multivariate analysis to define, differentiate, and classify the constituent peaks from the bacteria to detect clinically relevant information with precise specificity [65]. In a similar study, Pandey et al. used Raman spectroscopy to image myringosclerosis, a pathologic condition arising due to calcium deposits on the tympanic membrane, and to understand the molecular characteristics of these whitish, sclerotic plaques [66]. Similarly, Ayala et al. used an in vivo confocal assisted Raman microscope to analyze and study the profile of three commonly found bacteria, *Haemophilus influenzae*, *Moraxella catarrhalis*, and *Streptococcus pneumonia* grown on agar plates which are responsible for acute otitis media (AOM) [20]. Their group also conducted an extensive study on *Staphylococcus aureus* using Raman spectroscopy, to understand the antibiotic resistance capability of this species from the acquired Raman peaks [67]. In 2018, Boppart et al. developed and patented a Raman handheld device to detect microbiological constituents of the middle ear from the collected Raman spectrum [68]. This handheld Raman tool can serve as a point-of-care device providing the potential for label-free, non-invasive, real-time identification and detection of ear contagions.

**Figure 4:**
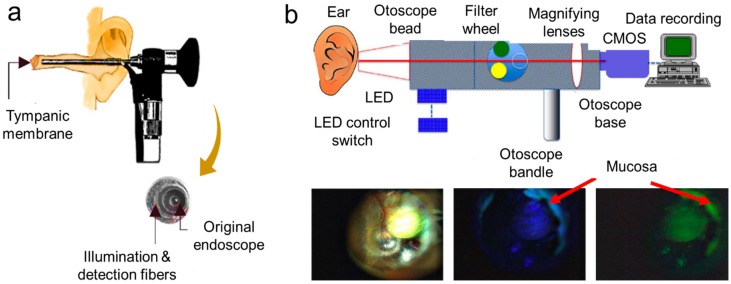
Tools available to detect middle ear infection. (**a**) Diffuse reflectance spectroscopy. Reproduced with permission from [68]. Copyright IOP Publishing, 2017. (**b**) Fluorescence otoscope. Reproduced with permission from [72], Copyright American Chemical Society, 2014.

Besides Raman spectroscopy, several other optical systems have been constructed lately. In this section, we present some of the notable works using emerging optical platforms for diagnosis of ear infection. For example, Sundberg et al. used diffuse reflectance spectroscopy, an infrared spectroscopy technique, to measure the hemoglobin content of the tympanic membrane in children to find whether or not they were affected with otitis media [69]. Figure 4a shows the schematic of their assembled setup, which included a probe head along with its illumination and detector fibers. Because the tympanic membrane is well supplied with blood vessels and sensory nerve fibers, the oxygenated hemoglobin peaks could be recorded at 542 nm and 576 nm, respectively. The hypothesis of their study was that the diffuse reflectance spectra from a healthy and an erythematous tympanic membrane ought to differ analogously with the spectra. This method is fast and non-invasive, making it very advantageous and competitive for in vivo measurements over other optical methods. However, their probe design may not be suitable for in vivo experiments for conscious children.

Scientists have also constructed ear phantoms to understand the acoustic transmission in the internal ear cavities. The middle ear cleft contains two capillary vessels: one with arterial blood takes away oxygen (O_2_), while the vessels with venous blood escalates the carbon dioxide (CO_2_) and water vapor (H_2_O) from the air at the fork. Because of the anatomy of the middle ear, this circulation is in the order of a few minutes such that the partial pressure of these gases in air and the blood vessels is equal [70]. This gas exchange mechanism has been used to explore different regions of the air-filled middle ear cavities such as the tympanic membrane caries, aditus, antrum, and the mastoid air cell system [71]. Recently, Zhang et al. developed a noninvasive optical method by combining reflectance and scattering absorption spectroscopy. In their setup, absorption spectroscopy was utilized for analyzing the gases by collecting the backscattered light through a fiber-optics probe, while the diffuse reflectance spectra were used simultaneously to determine the oxygen flow in the eardrum with a reflectance probe [9]. The combined spectroscopic approach was very convincing because the absorption marks of gases were nearly 10^4^ times finer compared to immediate solid constituents, providing a high accuracy of diagnosis. They recently improved their performance of detection by using a more realistic phantom compared to the previous studies, but clinical trials are yet to be performed in the near future [72].

**Figure 5:**
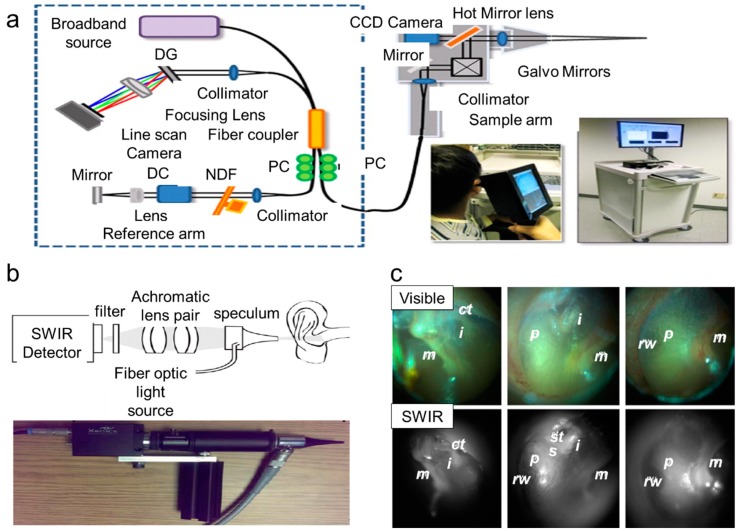
Tools available to detect middle ear infection. (**a**) Handheld OCT (optical coherence tomography) (diffraction grating (DG); polarization controller (PC); dispersion compensation (DC) materials; neutral density filter (NDF)). Reproduced with permission from [76]. Copyright Elsevier B.V.,2013. (**b**) SWIR (short wavelength infrared) otoscope. Reproduced with permission from [81]. Copyright National Academy of Sciences,2016 (**c**) Representative images for (**b**) Reproduced with permission from [81]. Copyright National Academy of Sciences,2016. ct, chorda tympani; i, incus; m, malleus; p, cochlear promontory; st, stapedial tendon; s, stapes; rw, round window niche.

Besides pneumatic assessment to differentiate healthy and diseased ear sections, based on single wavelength transmission, researchers have also explored multi-wavelength based imaging strategies. A few advantages of a multi-wavelength imaging system in comparison to the latter include the ability to obtain high-resolution cell images with reduced flare and low signal-to-noise ratio irregularities. For almost a century and to date, white light otoscopes were/are used extensively for the identification of external auditory canal and middle ear pathologies. Recently, Valdez et al. coupled the conventional otoscope with multiwavelength fluorescent filters and a CMOS camera to record videos of the ear infections. Figure 4b (top) shows the block diagram of their design, and Figure 4b (bottom) shows the images of the middle ear and tympanic membrane by using white light otoscopy and fluorescence imaging acquired at two different excitations (405 nm and 450 nm, respectively). Their team demonstrated this proof-of-concept platform for fluorescence imaging of congenital cholesteatomas (i.e., non-cancerous skin growth) found in the middle ear tissue. This assembled otoscope brought together a portable, adaptable, low-cost, feasible fluorescence imaging device for clinical identification of middle ear pathologies. However, their system was limited to the gain signal from the brightest chromophores, and it was difficult to get a proper signal-to-noise ratio for weak fluorescent features [73]. Studies have also been reported on applying non-contact optical sensing techniques or optical scattering techniques by using low-coherence light to obtain 2D/3D images of biological tissues/sections in micron resolution. This imaging technique is widely known as optical coherence tomography (OCT) or low-coherence interferometry (LCI). Nguyen et al. integrated these techniques to identify infected and non-infected ear based on light scattering phenomena arising from the middle ear biofilms [1]. Their research group also integrated LCI with a video otoscope to quantify the biofilms in an animal model based on the developed algorithm code [36]. The advantages of OCT are that it is non-invasive, causes no tissue damage, and is suitable for in vivo applications [74]. However, due to the long ear canal, sometimes, it is difficult to get a proper signal-to-noise ratio from the LCI/OCT setup as the beam focus is not able to reach there [1]. Near-infrared OCT has been demonstrated to detect middle ear biofilms by penetrating through the tissues of an ear with ultra-high-resolution up to nearly 1.3 μm and generating 3D images for visualization [75]. Jung et al. and Nguyen et al. were the forerunners in this area of research and also assembled a portable, handheld OCT system, as shown in Figure 5a. Their OCT setup contained a broadband source to generate low-coherence light, a fiber coupler for splitting two beams, and transferring it to the interferometer, a linear photodetector that captured the scattered beams, and a computer for data acquisition. They utilized this OCT system to detect bacterial biofilms accumulated in the eardrum. However, acquiring complete three-dimensional imaging is still a challenge for their system [76,77]. While a clinical otoscope only provided subjective or qualitative diagnosis, the assembled handheld OCT provided quantitative information about the biofilm progression in cases of middle ear infection. OCT has also been applied to understand AOM and chronic cases of otitis media in the tympanic membrane [75].Besides AOM identification, several studies have also utilized the handheld OCT for scanning biofilms from the tympanic membrane [1,77,78,79]. By combining a fiber based device with OCT, Boppart et al. studied possible reasons for the formation of fluid behind the ear drum as a result of otitis media with effusion [80].

The clinical otoscope has a single white light source only, which limits its applicability from an optical imaging standpoint. To overcome this, Carr et al. used short wave infrared (SWIR) light combined with an otoscope to analyze the anatomical structures situated after the thin tissue membranes inside the ear such as the ear drum [81]. SWIR covers the wavelength from 1.4 to 3 microns in the electromagnetic spectrum and as a result can often offer increased penetration and better image resolution that is otherwise not achievable with visible light imaging. In Figure 5b, we show the schematic of their SWIR system, which includes a fiber-coupled light source, a pair of achromatic doublet lenses, a disposable medical speculum, an indium gallium arsenide (InGaAs) sensor, and an SWIR detector. As shown in Figure 5c, they compared SWIR otoscope-acquired images with visible images and successfully identified the chorda tympani, cochlear promontory, malleus, stapedial tendon, incus, stapes, and round window niche (indicated by their initials as shown in the images). Even though their system was very much viable to image middle ear pathologies, sometimes it may require some supplementary training of the medical practitioners. In another similar study, Schaefer et al. also suggested an infrared light source for thermography for early recognition of various infections, not only in ears, but also other facial areas [82].

Confocal laser scanning microscopy (CLSM) is another emerging real-time in vivo imaging system. Hall-Stoodley used CLSM to obtain middle-ear mucosa biopsy specimens and concluded that otitis media was biofilm related [19]. On the other hand, Tang et al. used CLSM and identified three common bacteria, namely *H. influenzae*, *S. pneumoniae*, and *M. catarrhalis*, by using a fluorescent dye and a bacterial targeting ligand, Concanavalin A [83]. Similarly, Hoa et al. used confocal laser scanning microscopy (CLSM) to detect middle ear pathogens in biofilms obtained from the nasal cavities [84]. The bacteria type was identified by using type-specific 16S ribosomal RNA labeled with Cy3 and Cy5 fluorescent dyes, respectively. However, detecting biofilm in adenoid is a challenging task by using CLSM, and the role of adenoid biofilm in ear infection is very significant [84,85].

In recent years, more focus has been devoted towards building point-of-care devices/technology, for example handheld OCT or handheld Raman probes, for easy, handy, and accurate diagnosis in clinical or non-clinical settings [77,78,86].

## 4. Clinical Trials Using the Optical Imaging Techniques

Although the data in clinical trials are limited due to the tedious investigation protocols, number of subjects, and patient consent issues, we have listed a few published reports on Phase II clinical trials (in Table 3) wherein the number of patients/participants observed were greater than 10. We also categorized these approaches based on the device/technology used, brief objectives, and main findings. These clinical trials suggested that an otoscope is the most used medical device by healthcare professionals (e.g., physicians, nurse practitioners, audiologists) to screen or diagnose patients with ear infection followed by tympanometry and optical coherence tomography. 

## 5. Conclusions

This review summarized the literature regarding clinical and preclinical imaging techniques used for optical identification of middle ear infections. Clinical methods of investigating infections using a conventional otoscope, tympanometry, and optical coherence tomography were discussed along with their advantages and limitations. The list of clinical trial further presented the current medical devices used to diagnose middle ear infections. Furthermore, novel preclinical approaches and information on non-invasive Raman spectroscopy techniques for the detection of middle ear infection were presented to provide an outline of the current literature and to create a guideline for future progress. Although these non-invasive techniques are promising, future work should be directed to conducting clinical trials for these emerging imaging techniques to combat the suspected inefficiency in the current otologic diagnosis and help with the accurate treatment of middle ear infection decision making.

## Figures and Tables

**Table 1:** List of current and emerging optical techniques for the detection of ear infection. OCT, optical coherence tomography; AOM, acute otitis media.

Technology	Advantages	Limitations	Performance Parameters
Otoscope	Visually examines middle ear including coloration, transparency, and presence of liquid in the tympanic membrane.	Visual inspection may not be enough to detect the type of infection.	Sensitivity = 61–70%; Specificity = 61–70% [24,25].
Cell Scope	Visually examines middle ear like the otoscope with the help of a cellphone.	Visual inspection with the help of a cellphone may not detect the exact type of infection.	Sensitivity = 70%; Specificity = 70% [26,27].
Terahertz (THz) otoscope	Electromagnetic waves sensitively detect water molecules for feasible AOM diagnosis.	Terahertz waves are sensitive to membrane geometry.	* Sensitivity = 82–89%; * Specificity = 66–77% [28,29,30].
Acoustic reflectometry	Measure the fluid formation in the middle ear.	Limited information for structural changes in tympanic membrane.	Sensitivity = 63.6–96%; Specificity = 79.7–87% [31,32].
Tympanometry	Examine the movement of the eardrum by air pressure.	Structural changes in tympanic membrane cannot be detected accurately.	Sensitivity = 70–91%; Specificity = 71.7–98% [32,33,34].
Pneumatic otoscopy	Examine the mobility in the tympanic membrane.	Misinterpretation of diagnosis and often performed by untrained personnel.	Sensitivity = 74–94%; Specificity = 79–80% [25,35].
Raman spectrometer	Determine the unique chemical fingerprints of molecules responsible for ear infection.	Sometimes, Raman signals may need to be enhanced for better sensitivity of detection.	Sensitivity = 95.48%; Specificity = 99.06%.
Low-coherence interferometry (LCI) along with Raman scattering spectroscopy (RS)	Identify pathogens of ear infection.	Bacterial pathogens in body fluid cannot be detected.	Not available.
Low-coherence interferometry/optical coherence tomography	Non-invasive; causes no tissue damage, and suitable for in vivo applications.	Sometimes, the beam focus cannot reach to the long ear canal; thus, it delivers inadequate signal-to-noise ratio data.	Sensitivity = 68–86%; Specificity = 90–98% [1,36].
Diffuse reflectance spectroscopy	Measure the hemoglobin content of the tympanic membrane.	May not be suitable for in vivo experiment.	* Sensitivity = 89–100%; Specificity = 79–100% [37,38].
Reflectance and scattering absorption spectroscopy	Analyze the gases and also determine the oxygen flow in the eardrum.	Experiments were done on ear phantom and planning to be performed in clinical trials soon.	Not available.
Fluorescence otoscope	A platform for fluorescence imaging of congenital cholesteatomas (i.e., non-cancerous skin growth) found in the middle ear tissue.	Difficult to get proper signal-to-noise ratio for weak fluorescent features.	* Sensitivity = 96.7%; Specificity = 91.7% [39].
Hand-held OCT	Provide quantitative information about the biofilm progression in cases of middle ear infection.	Acquiring full three-dimensional in vivo imaging is difficult.	Sensitivity = 68–90.9%; Specificity = 90.2–98% [1,40]
SWIR (short wavelength infrared) otoscope	Analyze the anatomical structures situated after the thin tissue membranes inside the ear such as an ear drum.	Some supplementary training is required for the medical practitioners.	Sensitivity = *67–100%; Specificity = 89–100% [41].
Confocal laser scanning microscopy (CLSM)	Detect biofilm-related middle ear pathogens.	Detecting biofilm in adenoid is a challenging task.	* Sensitivity = 85.19–98.15%; Specificity = 97.6–99.26% [42,43].

* Refers to a similar technology, but not specifically applied to detect ear infection.

**Table 2:** Raman spectra and band assignments observed in common pathogens such as *S. pneumoniae*, *H. influenzae*, *P. aeruginosa*, *Moraxella*, *Streptococcus pyogenes*, and *Staphylococcus aureus* [58,59,60,61,64].

Range (cm^−1^)	Peak Assignment
640–675	Guanine (B-DNA), tyrosine valine
713–740	Adenine, glycoside
745–790	Cytosine, uracil, thymine, tryptophan
800–815	O–P–O (RNA)
930–990	C-C stretch (α-helix skeletal mode), C–N stretch
1000–1010	Phenylalanine, C–C aromatic ring stretch
1025–1060	C–C stretch (phospholipids, glucosidic rings), C–N stretch
1080–1105	PO^2−^/O–P–O (DNA), CO_3_^2−^/C–C or C–O–C stretching (carbohydrates)
1130–1145	C–O–C (unsaturated fatty acids)
1215–1295	Amide III (random), thymine phenylalanine, tryptophan
1330–1345	Adenine, guanine, C–H stretch
1390–1415	COO– symmetric stretch
1440–1475	CH_2_ deformation
1510–1560	Amide II (C=C)
1570–1595	Adenine, guanine (ring stretching), nuclei acid bands
1658–1700	Amide I
2890–2900	C−H-stretching deformation vibrations of CH_2_ and CH_3_

**Table 3:** List of Phase II clinical trials for ear infection detection.

Technology	Objective	Main Observations	No. of Patients Observed	Authors
Otoscope with cellphone: CellScope Oto (CSO)	○ Used for diagnosis of the tympanic membrane. ○ Handy, easy to use, good precision.	Physicians, patients, and parents favored CellScope Oto in comparison to the conventional otoscope as it was easy to use, had good diagnostic precision, with the benefit of image acquisition to track changes through the period of infection.	51 (adults)	Richards et al. [87]
SWIR otoscope	○ Used for examination of the tympanic membrane and to identify fluid accumulation in the middle ear. ○ The infrared transmission provides better visibility of the anatomical components of the inside ear.	SWIR facilitated non-invasive optical penetration to image deep ear tissues and identify fluid accumulation in the middle ear, which is otherwise difficult to visualize from a conventional otoscope.	10 (adults)	Carr et al. [81]
Otoscope	○ Used for routine checkup from parent-reported symptom for children with AOM (AOM-SOS).	Due to a lack of techniques to track early symptoms in children with AOM, a parent-reported AOM severity of symptoms (AOM-SOS) structured questionnaire was established to understand the AOM symptoms for better treatment trials.	264 (children)	Shaikh et al. [88]
Otoscope	○ Used to identify otitis media from endoscopic still images of the tympanic membranes.	To understand AOM diagnosis, endoscopic still images of the tympanic membrane were examined by expert otoscopists. Preventive antibiotic treatment was the individual-advised diagnostic criteria.	783 (children)	Shaikh et al. [89]
Pneumatic otoscope	○ Used for routine analysis to check for dullness or reflex from the tympanic membrane in newborns.	This study revealed the general ear features in healthy newborns (~ 72 h of life), mostly having pink/red colored eardrums, with a dull gray/opaque tympanic membrane.	81 (newborn)	Cavanaugh et al. [90]
Otoscope	○ Used to determine severity of AOM for both symptomatic and otoscopic conditions. ○ Routine analysis of tympanic membrane, bulla formation, hemorrhagic redness, and purulent effusion.	This clinical trial helped to understand the severity and symptoms of uni-/bi-lateral AOM in children aged 6 to 35 months. Assessment revealed that bilateral AOM was more severe than unilateral AOM.	232 (children)	Uitti et al. [91]
Tympanometry	○ Used to assess the effect of tympanometry on diagnosis of otitis media.	A randomized trial was conducted to understand physician diagnosis and prescription for OM when using either a tympanometry (specific to the middle ear) or an otoscope (sees all the ear). The study revealed that antibiotics were prescribed for OM in both cases.	698 (children)	Spiro et al. [92]
Tympanometry with otoscope	○ Used to compare the effectiveness of tympanometry with respect to pneumatic otoscopy.	The clinical trial study showed that tympanometry could be used as an adjunctive device with pneumatic otoscopy and not as a standalone device.	515 (children)	Helenius et al. [93]
Optical coherence tomography	○ Used for ear canal imaging of the human tympanic membrane to identify epithelial and collagenous layers and quantify their thickness.	The OCT clinical trials provided a non-invasive means to study the middle ear microstructure in vivo utilizing a safe near-infrared light source. Advantages include the ability to image diseased tissues with high resolution.	10 (adults)	Djalilian et al. [94]
Optical coherence tomography	○ Used for measuring the optical properties of the tympanic membrane. ○ Handheld, easy to use, for noninvasive routine analysis.	The biofilm thickness results from the OCT clinical trials revealed a statistically significant quantitative difference between normal, acute, and chronic otitis media (OM) infections.	34 (children)	Monroy et al. [24]
Combination of low-coherence interferometry and optical coherence tomography	○ For proper diagnosis and visualization of the tympanic membrane with and without biofilm growth.	The clinical findings from the OCT image scans in adults with chronic OM indicated the formation of biofilms as opposed to no biofilms in healthy subjects.	20 (adult)	Nguyen et al. [1]
Optical coherence tomography	○ Used to observe effusion of otitis media infected areas based on the axial depth scan.	Spectral domain-OCT (840 nm) was utilized to acquire axial depth scan images from normal and healthy ear to understand the OM infections. These OCT image databases could potentially serve as a means to upgrade the current otoscopic techniques.	39 (non-specified)	Cho et al. [95]
Confocal laser scanning microscopy (CLSM)	○ Used for identification of biofilm growth from otitis media and middle-ear mucosa (MEM) biopsy specimens.	The CLSM mucosal biofilm images, collected in this clinical study, revealed that chronic OM in humans is biofilm related.	26 (children)	Hall Stoodley [19]
